# A review of soft wearable robots that provide active assistance: Trends, common actuation methods, fabrication, and applications

**DOI:** 10.1017/wtc.2020.4

**Published:** 2020-09-14

**Authors:** Carly Thalman, Panagiotis Artemiadis

**Affiliations:** 1 Ira A Fulton Schools or Engineering, Arizona State University, Tempe, Arizona, USA; 2 Mechanical Engineering, University of Delaware, Newark, Delaware, USA

**Keywords:** soft robotics, wearable, exoskeletons, wearable robotics

## Abstract

This review meta-analysis combines and compares the findings of previously published works in the field of soft wearable robots (SWRs) that provide active methods of actuation for assistive and augmentative purposes. A thorough investigation of major contributions in the field of an SWR is made to analyze trends in the field focused on fluidic and cable-driven systems, prevalent and successful approaches, and identify the future direction of SWRs and active actuation strategies. Types of soft actuators used in wearables are outlined, as well as general practices for fabrication methods of soft actuators and considerations for human–robot interface designs of garment-like exosuits. An overview of well-known and emerging upper body (UB)- and lower body (LB)-assistive technologies is categorized by the specific joints and degree of freedom (DoF) assisted and which actuator methodology is provided. Different use cases for SWRs are addressed, as well as implementation strategies and design applications.

## 1. Introduction

A new and rapidly growing topic of research in wearable technologies is soft robotics. Over the past decade, assistive soft wearable robotic (SWR) devices have been advancing at an exponential rate, branching into numerous categories such as mobility-assistance achieving activities of daily living (ADLs), robot-facilitated therapy, and human augmentation, to name a few. In robotics, the term “soft” is typically used to describe materials that conform to existing surroundings and are compliant when exposed to external forces or interact with objects of unknown geometries. The use of materials that are inherently soft is conducive to the development of robotic systems that have demonstrated potential for safer, more comfortable, and lower cost alternatives when compared to rigid counterparts (Schiele and van der Helm 2009; Wehner et al. 2013). Research on soft robotics has proven that using such systems is effective for human–robot interaction, improved durability, and increased user comfort (Majidi 2013). It is a field with much promise and many novel technologies that are quickly beginning to change the way we think about wearable, assistive mechanisms (Majidi 2013; Bao et al. 2018).

The objective of this review is to analyze existing SWR devices in order to identify trends in the assistive robotics field and outline how a rapidly expanding topic has branched into different methodologies. This review presents a thorough look into how current research groups are aiming to assist the human body, collated by upper body (UB) and lower body (LB) categories. Trends of actuator methodologies used in the field are examined, and the benefits and drawbacks to each are identified for future investigation through a comprehensive analysis of recent work in this topic over the past decade.

## 2. Trends in Soft Wearable Robotics

### 2.1. Soft robotics in wearable assistive technology

Soft mechatronic technologies have become more reliable, effective, and well known over the last decade and have become an attractive solution for wearable robotic technologies (Bao et al. 2018). There has been a gradual advancement from rigid exoskeletons with isolated compliant methods of actuation toward a paradigm of entirely soft, garment-like wearables that feel nearly transparent to the user (Granberry et al. 2017). Wearable robotic devices made from soft, compliant materials have been gaining momentum in both academic and commercial settings over the past decade (Bao et al. 2018; Cianchetti et al. 2018). The lightweight, low-cost characteristics of garment-like devices forgo heavy, bulky, and rigid components in favor of inherently soft and compliant materials such as silicone elastomers, fabrics, and other forms of flexible materials (Coyle et al. 2018). The forgiving nature of the materials helps to alleviate issues with joint alignment that can be observed in some rigid exoskeletons (Schiele and van der Helm 2009) and provides safer interaction with people and the environment around the robotic system. The compliance of the materials can easily conform to its surroundings, the user’s joints, or the objects with which it interacts (Galiana et al. 2012). Soft robotics offers an unmatched level of flexibility and versatility, resulting in systems that mold easily to their surroundings and the user for increased comfort, safety, and ease of use.

### 2.2. Definitions, scope, and application

Some SWRs are entirely soft, monolithic, garment-like wearable devices, made from soft actuators, fabric, and other compliant materials, while other applications include minimal or reduced amount of rigid components as mounting points for the soft actuators. Categories of wearable robots can be simplified into four main groups as defined by Wearable Robotics Association (2015) and Exoskeleton Report (ExR) (2020): (a) military, (b) industrial, (c) consumer, and (d) medical. Most wearable devices aim to either provide assistance or augmentation. In this review, assistance is defined as an SWR with a goal of restoring a more “natural” movement of biomechanical behavior (Awad et al. 2017b) and serves a role in accomplishing ADLs (Spector and Fleishman 1998). Augmentation is defined as an SWR that applies active external forces to a healthy user to increase the capability, strength, and/or endurance of the said user.

In this review, SWRs are analyzed and sorted into upper body (UB) wearables or LB wearables categories. This review evaluates multiple types of actuator designs and approaches that provide active assistance. While the following sections briefly highlight the prevalence of passive types of assistance, this review focuses only on looking at SWRs that utilize active actuator assistance and therefore will exclude thorough examination or discussion regarding SWRs that rely on passive assistance. This review analyzes the two most prevalent and rapidly expanding methods of providing active power, actuation, and assistance in SWRs: cable-driven systems and fluidic actuation. While there are many other types of soft actuators being developed (as discussed in “Other types of actuation for SWRs” section), this review aims to break down and analyze some of the most widely explored actuator designs, methods, and applications for an SWR for cable-driven and fluidic actuation. Fluidic actuators are organized into subcategories based on the materials used in fabrication: McKibben/pneumatic artificial muscles (PAMs), elastomers, and textiles/fabric-based actuators. Each aforementioned actuator design will be discussed in greater detail in “Actuation methods and materials for SWRs” section.

### 2.3. Growing trends in assistive SWRs over the decade

**Figure 1.**
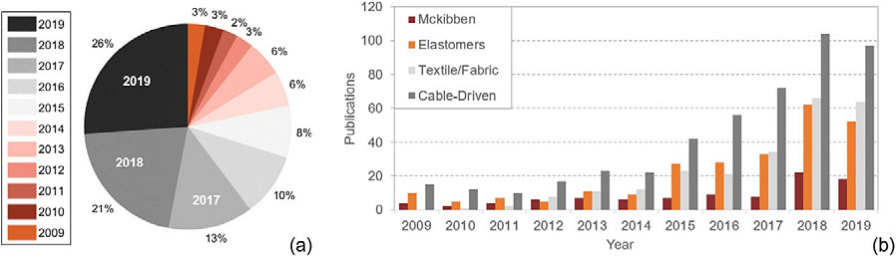
(a) Highlights the number of publications found on IEEE Xplore, including conference and journal publications, for research related to a soft wearable robot (SWR) between 2009 and 2019. Over the last decade, 57% of the existing research in an SWR has been published in the span of the last 3 years. (b) Indicates the number of publications found on IEEE Xplore, including conference and journal publications, for research related to an SWR between 2009 and 2019. Papers are refined according to type of actuation.

In order to determine how rapidly the interest in this sub-field of soft robotics has begun to grow, a meta-analysis of publications in this category is performed to analyze the rate of expansion. IEEE Xplore is used as the primary search engine, and all meta-data are searched for the specified conditions to determine the number of publications that fit the selected criteria. For this search, only peer-reviewed articles published as part of IEEE conference proceeding and journals listed in the IEEE Xplore search engine were included for the final publication count. The search was limited by year for each search, and the total number of publications was recorded from years spanning from 2009 to 2019, to obtain a record of relevant publications across the last decade. These data were collected on January 9th of 2020. The search parameters were limited articles that included the terms “soft” and “robot” in the title or main text and also containing the words “wearable” and “assist” in the metadata of the publications. The resulting information of the meta-analysis is shown in Figure 1a, which highlights that 60% of all publications on IEEE Xplore fitting the aforementioned criteria were published across 2019, 2018, and 2017, the last 3 years since this search was performed. These data indicate visible trends which show that soft robotics is itself a rapidly growing field, and additionally that research into an SWR has begun to expand at exponential rates in recent years. The last year evaluated (2019 from Figure 1a), shows the highest number of publications fitting within the confines of the refined search criteria, with 26% of the search results appearing in the most recent year.

To further evaluate the trends across SWRs published during this time, each search was refined in the respective year of publication by the actuation methods used (Figure 1b). These actuation methods, the pros and cons of each, as well as a more in-depth look, are included in the following sections. The increasing trend in publications and research interest can also be observed for each of the following actuation types, though it is notable that while cable-driven systems appear to remain predominant in most years, textiles and passive SWRs have begun to gain more popularity among published research. Where there were no notable SWR publications in 2009, this actuation method made up 16% of published work since 2017 and nearly 20% of the published SWR research in 2019. A push for rapid advancement in wearable technologies in the early 2010s may be partially attributed to military and government-funded projects in the field of robotics for more simplistic, lower cost, and lighter wearable devices to enhance human performance. Some of these major projects started with funding from DARPA and NASA grants with the Warrior Web (Asbeck et al. 2013) and the Armstrong (Kadivar et al. 2017). These exosuits aimed to reduce the overall metabolic cost to soldiers, who walk prolonged distances while carrying heavy loads, as well as reduce ergonomic issues and increase prevention of work-related injuries for astronauts.

## 3. Actuation Methods and Materials for SWRs

Fluidic actuators have been simplified and broken down into subcategories: McKibben/PAMs, elastomers, and textiles/fabric-based actuators. The pros and cons of each aforementioned actuator design will be discussed in greater detail in the following sections.

### 3.1. Development in fluid-driven soft actuators for SWRs

One of the earlier soft actuator design and most commonly implemented method of actuation used in the soft robotics field is the fluid-driven approach (Yariott 1972; Petersen and Shepherd 2018). This method uses fluidic pressurization of specific cavities within the soft actuators to entice some kind of motion or generate force and can be traced back to initial designs from the 1970s and early 2000s (Yariott 1972; Noritsugu 2005). Pneumatically powered systems use compressible fluids to generate variable stiffness within specific actuators. Compressed air is most commonly used due to low viscosity, high compressibility, accessibility, and low cost (Ding et al. 2014). However, pneumatic systems have suffered from issues with latency and controllability when expected to interact or react to the user’s kinematic or surrounding feedback (Malcolm et al. 2013; Ding et al. 2014). This review analyzes methods of soft actuation when applied to wearable devices, which are discussed in the following sections for McKibben/PAMs, elastomers, and textiles/fabric-based actuators.

#### 3.1.1. Pneumatic artificial muscles/McKibben

**Figure 2.**
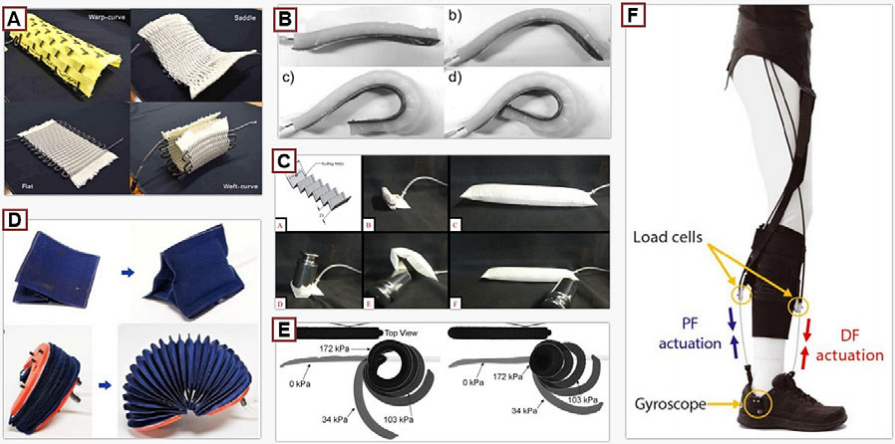
This figure provides a brief overview of different actuation methods used in soft exosuits: (a) Improved McKibben-type actuators braided into a mesh for higher contraction, lower profile, and a wider range of bending mechanics (Hiramitsu et al. 2019), (b) an elastomeric actuator that can be mechanically programmed to achieve different types of bending (Yap et al. 2015a), (c) a stiffening beam textile actuator designed to resisted bending and buckling (Miller-Jackson et al. 2019a), (d) new fabrication methodologies to create fabric-based inflatable actuators (Yang and Asbeck 2018), (e) shows fabric-based inflatables based on fiber reinforcement to induce different types of motions (Cappello et al. 2018a), and (f) a cable-driven soft wearable robot (SWR) device using Bowden cables to provide assistance (Awad et al. 2017b).

The PAM is one of the earliest developments in soft actuators for wearable devices, dating back as far as the 1970s for initial documentation (Figure 2a) (Yariott 1972; Klute et al. 1999). PAM actuators are typically characterized by a uniaxial contraction force upon actuator pressurization, which can be used to apply high tensile forces that closely resemble the mechanisms of human muscles contracting (Noritsugu 2005). When unactuated, PAMs have an intrinsic compliance from the soft materials that are nearly transparent to the user’s natural kinematics in comparison to a rigid exoskeleton. The McKibben muscle (or braided PAM) is a well-known PAM actuator (Yariott 1972; Klute et al. 1999; Davis et al. 2003) that began introducing the concept of using soft materials to create a robotic actuator for human assistance in the early 2000s (Noritsugu 2005; Kobayashi et al. 2007). McKibben muscles consist of a cylindrical shell (often a type of braided mesh) that is capable of radial expansion and becomes inextensible at a maximum threshold (Klute et al. 1999; Sasaki et al. 2005b). The shell contains an airtight, thin-walled rubber tube with hyperelastic properties and is capped on both ends with an inlet for pressurization (Yariott 1972). Once pressurized, the tube expands the radius of the shell, inducing an overall contraction in the PAM actuator and an increasing tensile strength (Majidi 2013). McKibben muscles have a high force-to-weight ratio which makes them a popular choice when assisting human joints that require high torque (Teng et al. 2012; Wehner et al. 2013). McKibben muscles can have low bandwidth for certain human movements (Wehner et al. 2012) and can cause discomfort related to friction from the constantly varying surface area when in contact with the body (Davis et al. 2003).

Some PAM actuators address these issues by decreasing the overall size for more effective operation (Hiramitsu et al. 2019) and increasing the types of motions that the actuators can achieve (Sasaki et al. 2005a; Hassanin et al. 2017; Funabora 2018). These new approaches are opening doors to biologically inspired exosuits that have the high force-to-weight ratio of a McKibben muscle (Bilodeau et al. 2018) but with less weight, bulk, lower operating pressures, and a higher degree of transparency and flexibility for the user (Abe et al. 2018; Hiramitsu et al. 2019).

#### 3.1.2. Elastomeric fluidic actuators

Wearable robotics began to embrace new types of soft actuators made from hyperelastic elastomers and other polymers shortly after the McKibben muscle began attracting researchers to using compliant actuators (Figure 2b) (Noritsugu et al. 2004; Noritsugu 2005). Elastomers and soft robotics were already the fields of research that were well in the process of becoming an emerging field, but it was not until the early 2010s that elastomeric actuators started maintaining a presence in the wearable robotics community (Petersen and Shepherd 2018). Initial investigations into using hyperelastic materials for actuator designs revealed functional implementation of using the actuators to assist the human hand (Noritsugu et al. 2004). Elastomeric actuators are forgiving when interacting with surroundings of a complex or unknown geometry (Galloway et al. 2013). The major advantage to systems of such high compliance is the simplistic, underactuated nature of the actuator, and successful examples in human assistance in the mid-2010s for rehabilitation have been showcased featuring silicone-based actuators (Oguntosin et al. 2015; Polygerinos et al. 2015; Wilkening et al. 2015). Due to the generally lower force output from fluidic elastomer actuators, a wide majority of applications have focused on assisting the hand or wrist but some focused on the lower limbs (Park et al. 2014b).

PneuNet actuators became a popular design choice for many researchers in an SWR following the launch of the open-source instructional website “Soft Robotics Toolkit” (Holland et al. 2014) which was released by Harvard University in 2014. This design hosts several individual hollow units or chambers formed in a single plane, connected by a central cavity to allow pressurization of all chambers (Mosadegh et al. 2014; Yap et al. 2015b). Strain-limiting material is embedded in the base of the actuator. Upon pressurization, the chambers begin to expand, and due to the difference in strain at the top of the expanding chambers and the constrained layer along the base, the actuator begins to curl (Polygerinos et al. 2015) Recent advancements utilizing elastomeric actuators center around applications where low forces and high compliance are needed. Elastomeric actuators in more recent designs have integrated multimaterial properties into a single actuator during the fabrication process of casting and curing silicone molds to generate a wide variety of actuator designs (Polygerinos et al. 2017). Other materials have been used to generate a wide range of motions (RoMs) and actions for each actuator, such as contraction (Low et al. 2016; Wirekoh and Park 2017; Han et al. 2018), bending or curling (Li et al. 2020), and expansion (Zhang et al. 2019a) for rehabilitative purposes generally focused on the hand, wrist, or a weakened ankle.

#### 3.1.3. Fabric-based inflatables and textiles

Many actuators used in SWR designs in recent years have switched to a fabric-based methodologies, using soft actuators made from fabrics and other forms of textiles and thin films to make soft exosuits more garment-like with reduced form factors (Figure 2c–e) (Granberry et al. 2017; Park et al. 2019b). Research has been conducted with fabric-based soft actuators since the early 2000s (Noritsugu et al. 2008; Yang 2017), even before the concept became widely popular to the SWR community. Thin-walled, airtight bladders made from binding two or more layers of the material together to form a pouch (Niiyama et al. 2015). Fabrics have been used to make soft actuators by using materials that bind together chemically with adhesive, thermally, or with ultrasound welding processes (Yap et al. 2017). The fabrics often do not possess the same hyperelastic properties as the silicone-based actuators, and so design considerations often revolve more heavily around geometric programming of materials (Khin et al. 2017). The benefits of using a single layer of thin film or textile is the simplicity in the final design, as well as the low profile the resulting actuator will possess when at atmospheric pressure. When depressurized, most textiles return to a state in which there is minimal volume or thickness in the actuator, which prevents the body of the actuator from becoming a hindrance to the movement of the user more so than rigid robotic counterparts (Chung et al. 2018). A combination of material properties, textiles, and basic geometric configurations of bladders is used to achieve the desired motion and force output (Felt et al. 2018; Cappello et al. 2018a).

New developments in fabric-based inflatable textiles have allowed for the implementation of new materials that can achieve a high variety of deformations and force translations (Cappello et al. 2018a; Realmuto and Sanger 2019). Fabric-based actuators have also been created to mimic the behavior of bellows to create a bending or rotary motion, similar in mechanical principles to the PneuNet actuator (Khin et al. 2017; Felt et al. 2018; Thalman et al. 2018; Yang and Asbeck 2018; Miller-Jackson et al. 2019b). Similar to fiber-reinforcing elastomers, design and characterization of fabric actuators can be performed by combining inextensible fabrics with extensible fabrics, such as spandex or neoprene to obtain the desired actuator behavior (Cappello et al. 2018a; Realmuto and Sanger 2019). Other common methods of fabrication include heat-welded thermoplastic polyurethane (TPU) (Niiyama et al. 2015) and fabric-based inflatables made from weather-proof nylon or women mesh (Yap et al. 2017; Abrar et al. 2019; Thalman et al. 2019). Some fabric-based designs and textiles are striving to create smart garments and have developed sheets of fabric controlled through pneumatics (Funabora 2018; Hiramitsu et al. 2019).

### 3.2. Cable driven

Designing new, lighter weight, lower profile wearable robots began to converge toward main-streamed popularity around the early 2010s as the term “exosuit” became more well-known and defined (Figure 2f) (Asbeck et al. 2014). While PAM actuators had been effective in providing assistance, SWRs rapidly gained attention from researchers with the introduction of cable-driven mechanisms, which aimed to address several issues observed in early developments of fluidic SWRs (Galiana et al. 2012; Asbeck et al. 2013). Cable-driven actuation (Figure 2f) can easily mimic behaviors of the human musculoskeletal system to assist specific motions (Asbeck et al. 2014; In et al. 2015; Awad et al. 2017b; Zhang et al. 2019b). These systems have developed significantly in the past decade and are still one of the more prevalent actuation methods used. Extensive research has been done in controllability (Ding et al. 2018), anchor points for applied loads (Yandell et al. 2020), and design (Kwon et al. 2019) to create high performing exosuits.

The cables (or tendons) are affixed to a point on the exosuit (typically a strap or tab made from inextensible synthetic fabrics or lightweight plastics) which interfaces with the human body to allow for proper force transmission to the targeted joint (Asbeck et al. 2014; In et al. 2015; Awad et al. 2017a). The cable is routed along the body and fed to a motor which, when activated, winds the cables to apply a tensile force to the anchor point to mimic the musculoskeletal behaviors of specified joint or area (Awad et al. 2017b). Selecting an ideal anchor point is critical to ensuring the forces are translated to the body safely and effectively, as misalignments can cause the SWR to underperform (Galiana et al. 2012; Asbeck et al. 2014). Actuator power is transmitted to the direct point of contact (Yandell et al. 2017) by engaging the motors or pulley systems to provide varying levels of tension and movement on each cable. Sheathing is often used to help reduce friction of the cables moving across the skin and reduce overall friction (Nilsson et al. 2012). The interface that attaches the cable to the user must avoid slippage, allow for predictable torque generation when the cable is tensioned, and be comfortable to the wearer (In et al. 2015; Asbeck et al. 2015b). Cable-driven actuation can produce high torques at a bandwidth higher than that of other methods of soft robotic actuation used with SWRs. As a result, cable-driven systems are a popular approach for augmentation of performance (Quinlivan et al. 2015). However, such high tensile forces applied at singular points on the body can cause discomfort (Schiele 2009; Quinlivan et al. 2015; Baltrusch et al. 2018).

### 3.3. Comparison and critical evaluation of actuation methods in SWRs

**Figure 3.**
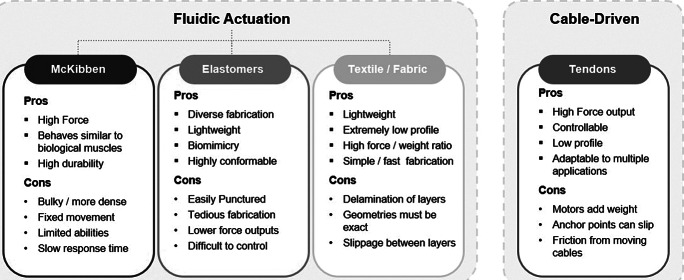
Brief overview of different actuation methods used in soft exosuits and the benefits/disadvantages of the listed types.

Each type of actuator has benefits and drawbacks depending on the application and the user. Designing an effective SWR means being able to select the materials and methods needed for the chosen application and ensure the selection will sufficiently meet the need. Listed in this section is a summarized overview of some of the pros and cons of each type of actuator. The highlights of each section are outlined in Figure 3, which categorizes the actuation methods discussed in the previous sections.

*Pneumatic Artificial Muscles (McKibben)*:

- **Pros**—McKibben muscles have a higher force/weight ratio and are able to withstand and generate higher forces during pressurization than other pneumatic designs. The actuators behave similarly to the human muscle, which simplifies design conceptualization and can easily provide assistance (Wehner et al. 2013). The lightweight and flexible nature of the inactive actuator make it easy to integrate onto a person without adding excessive weight to the users. The design of the braided mesh McKibben muscles is durable and can withstand the wear and tear of daily and repeated use.
- **Cons**—McKibben actuators face some issues with bulkiness and anchoring points (Kobayashi et al. 2007). Some McKibben muscles can be large in diameter to facilitate the needed force output, and the anchoring point of the point load from the actuator must be securely fixed on the user in order function as anticipated.
*Elastomeric Fluidic Actuators*:
- **Pros**—Elastomeric actuators can achieve high degrees of freedom with a single input and are forgiving against complicated joints or surfaces. Elastomers are less impacted by misalignment between the actuator and joint when subjected to an injection of pressurization and conform easily to their surroundings without additional input. Actuators are made from lightweight materials that have hyperelastic properties and expand and interact over larger surface areas against the body to avoid anchor point issues. This is useful for assistance to joints where additional bulkiness or anchor points may restrict user movement. These actuators can also reach a high degree of biomimicry which can be helpful when designing actuators to perform a specific biological or kinematic task.
- **Cons**—Elastomeric actuators begin to see a disadvantage in the aspect of controllability, due to the nonlinear behaviors exhibited upon pressurization and the general lack of sensing capabilities for position or control. Slower actuation time can be a limiting factor in the final application and higher force output can lead to inevitable structural failure, which would not be ideal for tasks that require higher force or cyclical repetition during assistance (i.e., heavy lifting or walking). Tethers to the pneumatic source may also be limiting to user motions or system portability.
*Fabric-Based Inflatables and Textiles*:
- **Pros**—Textile-based actuators have similar benefits to elastomeric alternatives; however, textiles are even lighter weight and can achieve a nearly undetectable profile when not pressurized (Yang and Asbeck 2018). Textile actuators can achieve higher force output at elevated pressure levels with inextensible fabrics, which increases the strength of the material properties to match the most durable inextensible layers. Textile or fabric actuators motion is predetermined by the materials and the corresponding material properties selected during fabrication (Cappello et al. 2018a). Fabrication techniques are easily accessible and simple, and design possibilities are limited mainly by the materials present or readily available for development (Yang and Asbeck 2018).
- **Cons**—Textile-based actuators face the same limitations as the aforementioned fluidic actuators with power source portability and latency. Other disadvantages can arise from mechanical fragility or failure, delamination of layers which create fluidic chambers, and slippage between layers of fabric and the human (Cappello et al. 2018a).

*Cable Driven*:

- **Pros**—The pros of cable-driven systems are low profile, lightweight, high torque, and increased controllability when compared to fluidic actuators. Highly complex motions can be achieved with a simple and underactuated input (Asbeck et al. 2013). This is beneficial for joints such as the hand or the shoulder or assisting multiple joints in series, such as LB limbs during walking. High torque is also easily achievable and controlled through fine-tuned input to the actuator motors, which allows for fast response time at precise and specified values. Cable-driven systems do not require a tether and are highly portable which is helpful to ADL tasks dealing with locomotion.
- **Cons**—Some drawbacks of cable-driven systems are friction, backlash hysteresis, nonlinearities (Dinh et al. 2017), and force translation to anchor points on the user (Galiana et al. 2012). Force and torque translation from the tendons to the joints relies on applying a point load to a soft or flexible interface (often garment-like exosuits or braces) which can result in slippage or hysteresis (Dinh et al. 2017). Slippage can often be observed between the skin and the fabrics, which can cause discomfort or inaccurate actuation based on nonobservable discrepancies with this displacement. The user typically must support the weight of the motors and other supporting hardware on their body (Asbeck et al. 2013).

### 3.4. Other types of actuation for SWRs

The following are actuation methods and designs that are beginning to be of more interest in the field of SWRs but have not yet become as effective or thoroughly investigated at this stage. These actuation methods have proven highly effective when implemented properly and are therefore worth noting. However, these actuator designs and methodologies are not within the scope of the review paper and will not be discussed in depth beyond this section.

Origami-inspired actuators are made from very thin, lightweight materials and designed to fold or bend in ways that mimic soft materials (Li et al. 2017; Sedal et al. 2018). Actuators can be controlled via small motors or are sometimes placed inside sealed thin film structures. Negative pressure can then be applied to remove the air from the thin film casing, forming a reinforced, folded structure encased inside the vacuum chamber contained in the thin film. Negative pressure can often be used just as effectively as positive pressure (Felt et al. 2018), which can be used in combination with other soft materials (i.e. foam, sand, paper, etc.) which occupy a certain volume at atmospheric pressure and compress and become stiff when subjected to negative pressure (Robertson and Paik 2017). Another form of actuation method used is the shape-memory alloy (SMA) actuator, which uses electrical current to affect the behavior, shape, and/or length of a material used in designing an actuator (Park and Park 2019). Another method is passive assistance from flexible or relatively soft materials. These actuators use materials with some intrinsic compliance or elasticity, to provide passive assistance to the user (Higuma et al. 2017; Lee et al. 2017b).

## 4. Actuator Designs for SWRs

**Figure 4.**
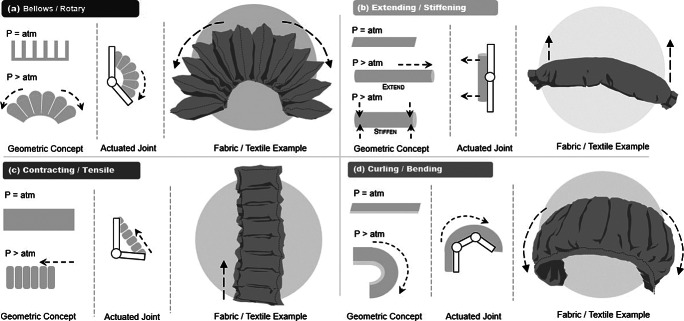
This figure provides an overview of the most commonly used actuator designs in soft wearable robots (SWRs) and the driving mechanical principles behind the motions supported by each listed category. Categories have been broken down into (a) bellows/rotary, (b) extending/stiffening, (c) contracting/tensile, and (d) curling/bending designs. Each design is illustrated as a geometric representation of the actuator before and after injection of pressurized air, the actuator acting on a conceptual joint, and an illustration of a fluidic textile representation of these actuators for reference.

Initial characterization of functional constraints for SWRs design requires initial identification of the actuation methods selected, which joint(s) will be assisted, and how the forces will be translated from the actuator to the user to provide effective, comfortable, and predictable assistance (Yandell et al. 2020). In most cases, the actuator is designed around a particular type of assistance that is needed at a selected joint on the human body or motion that is targeted for improvement. This can be done by utilizing actuators with different types of functionality (Connolly et al. 2015; Wang 2016). Throughout some of the most commonly seen SWRs, there are themes of the actuator designs designated for particular types of motion assistance and deformation methods. These were touched on briefly in “Cable driven” section but were more specific to elastomeric actuator design as an isolated concept. This section aims to highlight overarching themes of soft actuator designs commonly used in SWRs, specifically focused on fluidic actuators. These actuator designs have been categorized as shown in Figure 4 by (a) bellows/rotary, (b) extending/stiffening, (c) contracting/tensile, and (d) curling/bending designs.

### 4.1. Bellows/rotary

The first design in Figure 4a builds upon the PneuNet actuators described in “Cable driven” and “Comparison and critical evaluation of actuation methods in SWRs” sections, such as works presented at Harvard University (Polygerinos et al. 2015) and Arizona State University (Thalman et al. 2018), for elastomeric- and textile-based actuators, respectively. This design utilizes individual chambers or pockets that expand when inflated, as shown in Figure 4a. The base has a strain-limiting layer while the top is free to move. This allows for expansion across the top surface, while expansion is restricted at the base. Each chamber expands and exerts a contact force against adjacent chambers, which results in an effect that resembles bellows and generates a rotational motion (Miller-Jackson et al. 2019c). This actuator design is effective for assisting joints in a single degree of freedom (DoF) and has been commonly used in flexion of joints (such as the elbow or a finger).

### 4.2. Extending/stiffening

The design forms a straight beam-like shape which will either stiffen or extend depending on the exact design and intended purpose (Figure 4b). Creating a beam of variable stiffness can allow for corrections in kinematics and support based on the torque and stiffness provided by the beam (Miller-Jackson et al. 2019a). This actuator design can provide variable stiffness as seen in the elastomeric design from National University of Singapore (Koh et al. 2017), where the inherent stiffness and recoiling effect of the beam are used to push the elbow into extension. Another example is the ExoBoot from Harvard University (Chung et al. 2018), which uses an inflatable textile beam to push the foot into plantarflexion. The beam design is beneficial when either placed laterally across a joint to serve as a split or pull into alignment when activated (Natividad and Yeow 2016) or is placed in acute angle of a joint to push the joint to straighten as the beam inflates and extends (Miller-Jackson et al. 2019c). This design is most commonly used in joint extension or joint bending prevention (e.g., finger or knee).

### 4.3. Contracting/tensile

This actuator design is the most common in exosuits, mainly because it includes cable-driven systems by nature, as well as McKibben muscles. Actuators that generate tensile forces mimic concentric contraction of human muscles, which provides many design concepts for SWRs that behave similarly to natural biological functions. Cable-driven actuation functions similarly to tendons in the body, and fluidic contracting actuators are designed to contract like muscles when an internal pressure is applied as depicted in Figure 4c. Researchers at Carnegie Mellon developed an elastomeric version of a PAM that generates a tensile force when pressurized (Wirekoh and Park 2017), and a fabric-based contracting actuator was developed from Arizona State University (Thalman et al. 2019). Contracting actuators are the most widely seen design and support joints in all motions from flexion to extension and are commonly seen assisting multiple joints in series (ankle + hip, fingers + hand, etc.).

### 4.4. Curling/bending

This design (depicted in Figure 4d) is in a separate category from the bellows/rotary design to distinguish the different methods in which this motion is achieved. Specifically, the materials used and the properties that interact when subjected to internal pressure or load will generate the resulting deformation. The top layer is made from a hyperelastic or highly extensible material, while the base layer is made from or constrained with an inextensible material. This difference in extensibility will cause the actuator to curl around the inextensible layer when pressurized (Noritsugu et al. 2008). The benefit of an actuator that can curl rather than rotate about a point is the forgiving nature, which can easily form around complex joints with a single input. This design is commonly placed on the outside of a joint, such as the finger to assist flexion. This is seen in the three discussed fluidic actuation methods with modified McKibben muscle from University of Salford (Hassanin et al. 2017), elastomeric design (Yap et al. 2015a), and fabric-based design from Harvard University (Cappello et al. 2018b).

## 5. Fabrication Methods for Soft Fluidic Actuators

The McKibben muscle (or braided PAM) is a durable soft actuator that is made from a single hollow tube, which is impermeable to air (Yariott 1972; Klute et al. 1999). This tube is inserted into a braided mesh tube of a slightly larger diameter. The braided mesh moves as able to slide and expand radially up until a certain point where it becomes inextensible. This braided mesh restricts the inner tube from overexpanding or weakening/bursting if overinflated. It also allows for the inner tube to inherit the material properties of the braided mesh when fully radially expanded which increases the strength of the actuator (Klute et al. 1999; Sasaki et al. 2005b). The ends of the layered tubes are capped with a metal ring or other airtight fitting that allows for a tube to connect. When pressurized, the tube expands inside the braided mesh, which experiences a radial expansion that results in a reduction in overall actuator length (Yariott 1972). This is the governing principle behind the McKibben muscle actuator, and it is a design that has managed to stay relevant through the past decade due to the versatility, low cost, and easy of fabrication (Noritsugu 2005).

Elastomers (or elastic polymers) typically have a low Young’s modulus. A metric to measure the hardness of these materials is commonly identified using the Shore Hardness Scale, which can help determine how inertly stiff or flexible a material will be prior to actuator fabrication and during analytic and Finite Element Analysis (FEA) modeling (Holland et al. 2017). Elastomeric, fluidically actuated technologies have an extremely versatile RoMs, in categories of deformation types such as (a) bending, (b) expanding, (c) contracting, (d) elongating, and (e) twisting (Connolly et al. 2015; Wang 2016; Schmitt et al. 2018). These movements can be mechanically programmed into the actuator during the fabrication process (Galloway et al. 2013). Inextensible components are embedded within the elastomer to limit the strain at certain points and restrict expansion of the material during fluidic pressurization (Wirekoh and Park 2017; Heung et al. 2019). Fiber-reinforcement provides predictable, programmable motions depending on the orientation and positioning of the inextensible fibers (Galloway et al. 2013; Connolly et al. 2015). Strain-limiting layers such as a mesh or fabrics can be cured within the casting process to achieve a similar programmable motion (Polygerinos et al. 2015). For silicone-based elastomers specifically, there is a casting process that allows for complete customization and has been thoroughly explained and detailed for research groups and individuals to attempt independently (Holland et al. 2014).

Fabrics and textile-based actuator designs typically involve creating a specified, predetermined shape for a chamber and thermally or chemically bonding two or more layers of material together to form an airtight seal with a net shape of the desired chamber (Niiyama et al. 2015). Some actuators use only one type of material to achieve complete actuator fabrication (Yang and Asbeck 2018). They can be constructed from a heat-sealable material such as TPU, which forms an airtight seal when consistent pressure and heat are applied to form a thermal bond between layers (Niiyama et al. 2015; Thalman et al. 2018; Ang and Yeow 2019). Actuators may be formed from just TPU (or other heat-sealable thin films) layers (Niiyama et al. 2015), or they may use a fabric coated on one or both sides with a layer of heat-sealable film (Yang and Asbeck 2018; Abrar et al. 2019; Thalman et al. 2019; Park et al. 2019b; Miller-Jackson et al. 2019c). As soft components are ideally lightweight and compact, actuators are designed to produce the highest forces possible with the smallest associated volumes (Coyle et al. 2018; Cappello et al. 2018a). New manufacturing methods optimize this process and reduce the time and level of complexity involved in fabrication (Miller-Jackson et al. 2019c). FEA studies have shown promise in providing realistic models for actuator design and optimization by simulating the behavior of soft materials (Holland et al. 2017; Coyle et al. 2018). This can allow for easier material selection and geometric optimization for mechanical programming of soft materials (Polygerinos et al. 2015). Automated methods of manufacturing are being developed utilizing custom three-dimensional (3D) printers for additive manufacturing using soft materials and Computer Numerical Control(CNC) methods of heat sealing textiles to increase complexity and accuracy of actuator designs while simplifying the labor-intensive portions of actuator fabrication (Niiyama et al. 2015; Ang and Yeow 2019; Park et al. 2019b). Another method of creating these seams between layers is by using ultrasonic welding, which has successfully produced fabric-based actuators in previous work (Yap et al. 2017).

### 5.1. Advanced fabrication methods used in SWR Actuators

Some exosuits utilize smaller rigid or semi-rigid components as an integral part of the design, to create “anchor points,” which relieve load on the wearer (Asbeck et al. 2013; Quinlivan et al. 2015; Li et al. 2018). By integrating anchor points correctly, strength and structure can be provided to the device without sacrificing flexibility and wearability for the user (Jiang et al. 2018). Rigid components are kept a safe distance away from the user, ensuring that the user only directly interacts with soft components (Balasubramanian et al. 2008). Rigid or semi-rigid components can also be embedded in the soft exosuit, used to help translate large forces more comfortably across the body and provide minor structural support within the exosuit (Quinlivan et al. 2015; Li et al. 2018).

Fabrication methods of soft exosuit devices are myriad and evolving at a comparable rate to the evolution of the designs themselves (Miller-Jackson et al. 2019c). New fabrication methods, and the developments that they facilitate, include the use of sew-free textiles (Yang and Asbeck 2018; Connolly et al. 2019), soft lithography for stretchable electronics, and the use of multimaterial 3D printers to integrate several different soft materials into the same part (Yap et al. 2016). In sew-free anisotropic textiles, a film is applied to the desired textile material to make it air impermeable (Connolly et al. 2019). Then, a water-soluble polymer is applied alongside a heat press to bond the seams in an air-impermeable way, and any excess polymer can be dissolved using water (Connolly et al. 2019). This eliminates the need for complex seamwork using traditional sewing techniques, allowing for faster turnaround and less technical skill required on the part of the manufacturer. It also allows for new materials that otherwise would not be feasible for soft robotics, like anisotropic textiles, to create actuators that exhibit anistropic properties (Connolly et al. 2019).

## 6. Upper Body Soft Assistive Robotics

**Figure 5.**
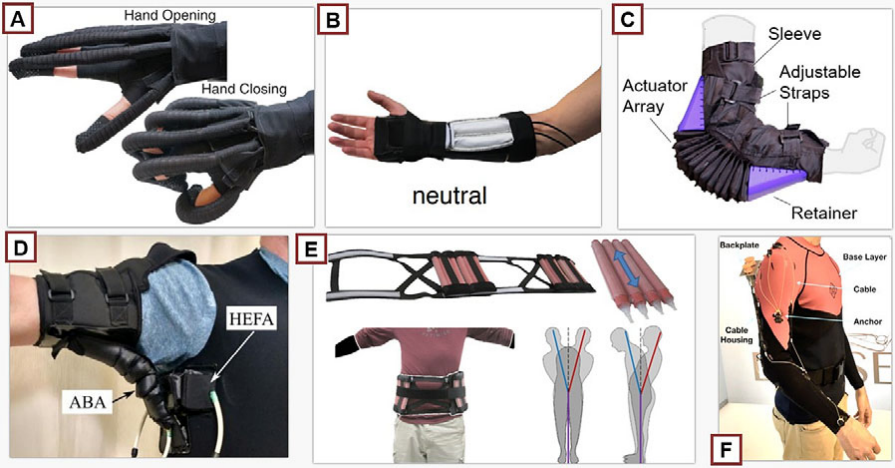
A brief survey of recent soft wearable robot (SWR) technologies for each of the following upper body joints: (a) A soft robotic hand developed at Harvard University at the Wyss Institute for Biologically Inspired Engineering (Cappello et al. 2018b), (b) a soft assistive device for the wrist made from fabric materials (Realmuto and Sanger 2019), (c) a soft elbow exosuit designed at Arizona State University (Thalman et al. 2018), (d) shoulder-assistive device also from Harvard University at the Wyss Institute for Biologically Inspired Engineering (O’Neill et al. 2017), (e) trunk orthosis from the Reconfigurable Robotics Lab at Ecole Polytechnique Fédérale de Lausanne (Robertson and Paik 2016), and (f) an upper body device that assists multiple joints via cable-driven actuation (Lessard 2018).

UB assistance often focuses more on stationary rehabilitation, assistance in achieving the ADLs, or injury and fatigue prevention (Maciejasz et al. 2014). Figure 5 presents a brief look at some successful UB soft robots assisting various joints, and Tables 1 and 2 provide a more comprehensive list of some of the more recently developed technologies listed in this section. The UB has a high level of complexity and many DoF isolated to specific joints in concentrated areas that are easily affected by additional weight (such as the hand). The benefits of using SWRs for UB assistance avoid placing heavy components or restricting joint alignments on the user’s arms or torso. Many of these SWRs for the UB are outlined in the following sections to discuss several types of assistance in more detail.

**Table 1.** This chart illustrates the correlation between existing soft wearable robots (SWRs) for the upper body, showing the most prevalent method of actuation used related to the specific type of assistance provided at each joint of the upper body and the respective degrees of freedom that are assisted.

Joint	Year	Soft wearable robot	Actuation	Method	Design	DoF	Motion
a. Shoulder	2008	Balasubramanian et al. (2008)	Pneumatic	McKibben	Contraction	2	Ab, C
	2019	Abe et al. (2019)	Pneumatic	McKibben	Contraction	1	F
	2012	Galiana et al. (2012)	Cable-driven	Bowden cables	Tensile	1	Ab/Ad
	2014	Koo et al. (2014)	Pneumatic	Textile/fabric	Rotary	1	Ab
	–	–	Cable-driven	Bowden cables	Tensile	1	F/E
	2016	Natividad and Yeow (2016)	Pneumatic	Textile/fabric	Stiffening beam	1	Ab
	2017	Kobayashi et al. (2007)	Pneumatic	McKibben	Contraction	3	F/E, Ab/Ad, C
	2017	O’Neill et al. (2017)	Pneumatic	Textile/fabric	Rotary	2	Ab/Ad
	–	–	Pneumatic	Textile/fabric	Stiffening beam	2	F/E
	2017	Lessard et al. (2017)	Cable-driven	Bowden cables	Tensile	2	F/E, Ab/Ad
	2018	Abe et al. (2018)	Pneumatic	McKibben	Contraction	2	F/E, Ab/Ad
	2018	Han et al. (2018)	Pneumatic	Elastomeric	Extension	2	F, Ab
	2018	Lessard (2018)	Cable-driven	Bowden cables	Tensile	2	F/E, Ab/Ad
b. Trunk	2015	Ohno et al. (2015)	Pneumatic	McKibben	Contraction	1	E
	2016	Inose et al. (2016)	Pneumatic	McKibben	Extension	1	E
	–	–	–	Textile/fabric	Stiffening	1	E
	2016	Robertson and Paik (2016)	Pneumatic	Elastomeric	Bending	2	F/E, LF
	2017	Cho et al. (2017)	Pneumatic	McKibben	Extension	1	E
	–	–	–	Textile/fabric	Stiffening	1	E
	2019	Yang et al. (2019)	Cable-driven	Bowden cables	Tensile	1	F
c. Elbow	2005	Noritsugu (2005)	Pneumatic	McKibben	Contraction	1	F
	–	–	–	McKibben	Bending	1	E
	2005	Aragane et al. (2008)	Pneumatic	Textile/fabric	Bending	1	F/E
	2014	Koo et al. (2014)	Cable-driven	Bowden cables	Tensile	1	F
	2015	Nycz et al. (2015)	Cable-driven	Bowden cables	Tensile	1	F/E
	2015	Wilkening et al. (2015)	Pneumatic	Elastomeric	Rotary	1	F/E
	2017	Xiloyannis et al. (2017)	Cable-driven	Bowden cables	Tensile	1	F/E
	2017	Koh et al. (2017)	Pneumatic	Elastomeric	Bending	1	F
	–	–	–	Textile/fabric	Stiffening beam	1	E
	2018	Tripanpitak et al. (2018)	Pneumatic	McKibben	Contraction	1	F
	2018	Gao et al. (2018)	Pneumatic	Elastomeric	Bending	1	F
	2018	Thalman et al. (2018)	Pneumatic	Textile/fabric	Rotary	1	F
	2018	Chiaradia et al. (2018)	Cable-driven	Bowden cables	Tensile	1	F/E
	2018	Li et al. (2018)	Cable-driven	Bowden cables	Tensile	1	F/E
	2018	Lessard (2018)	Cable-driven	Bowden cables	Tensile	1	F/E
	2019	Irshaidat et al. (2019)	Pneumatic	McKibben	Contraction	1	F
	2019	Abrar et al. (2019)	Pneumatic	Textile/fabric	Extending beam	1	E
	–	–	Cable-driven	Tendons	Contraction	1	F
	2019	Miller-Jackson et al. (2019c)	Pneumatic	Textile/fabric	Rotary	1	F
	–	–	–	Textile/fabric	Stiffening beam	1	E
	2019	Abe et al. (2019)	Pneumatic	McKibben	Contraction	1	F

Abbreviations: Ab, abduction; Ad, adduction; C, circumduction; E, extension; F, flexion; LE, lateral flexion.

**Table 2.** Continuation of Table 1, showing soft wearable robots (SWRs) for the upper body, specifically focused on the hand and wrist as this is an area that has been thoroughly explored by the listed groups using the actuation types specified in the following table.

Joint	Year	Soft wearable robot	Actuation	Method	Design	DoF	Motion
d. Wrist	2005	Noritsugu (2005)	Pneumatic	McKibben	Bending	1	Ab/Ad
	2005	Sasaki et al. (2005a)	Pneumatic	McKibben	Contraction	2	F/E, Ab/Ad
	2008	Balasubramanian et al. (2008)	Pneumatic	McKibben	Bending	2	E, Su
	2017	Lessard et al. (2017)	Cable-driven	Bowden cables	Tensile	1	Su/Pr
	2017	Hassanin et al. (2017)	Pneumatic	McKibben	Bending	1	Ab/Ad, Su/Pr
	–	–	–	–	Contraction	1	Ab/Ad
	2017	Zhu et al. (2017)	Pneumatic	Textile/fabric	Bending	1	F/E
	2018	Lessard (2018)	Cable-driven	Bowden cables	Tensile	1	Su/Pr
	2018	Li et al. (2018)	Cable-driven	Bowden cables	Tensile	1	Su/Pr
	2019	Ang and Yeow (2019)	Pneumatic	Elastomeric	Rotary	1	F/E, Su/Pr
	2019	Park et al. (2019b)	Pneumatic	Textile/fabric	Contraction	1	Su/Pr
	2019	Realmuto and Sanger (2019)	Pneumatic	Textile/fabric	Bending/curling	1	Su/Pr
e. Hand	2004	Noritsugu et al. (2004)	Pneumatic	McKibben	Bending	1	F
	–	–	Pneumatic	McKibben	Extension	1	E
	2008	Noritsugu et al. (2008)	Pneumatic	Textile/fabric	Bending	1	F
	2012	Nilsson et al. (2012)	Cable-driven	Bowden cables	Tensile	1	F
	2014	Lee et al. (2014)	Cable-driven	Tendons	Tensile	1	F/E
	2015	Yap et al. (2015b)	Pneumatic	Elastomeric	Rotary	1	F
	2015	Yap et al. (2015a)	Pneumatic	Elastomeric	Bending	1	F/E
	2015	In et al. (2015)	Cable-driven	Bowden cables	Tensile	1	F/E
	2015	Nycz et al. (2015)	Cable-driven	Bowden cables	Tensile	1	F/E
	2015	Polygerinos et al. (2015)	Pneumatic	Elastomeric	Bending	1	F
	2016	Borboni et al. (2016)	Cable-driven	Bowden cables	Tensile	1	F
	2016	Zhao et al. (2016)	Pneumatic	Elastomeric	Tensile	1	F/E
	2017	Meeker et al. (2017)	Cable-driven	Bowden cables	Tensile	1	E
	2017	Xiloyannis et al. (2017)	Cable-driven	Bowden cables	Tensile	1	F/E
	2018	Cappello et al. (2018b)	Pneumatic	Textile/fabric	Bending	1	F/E
	2018	Kang et al. (2018)	Cable-driven	Bowden cables	Tensile	1	F/E
	2018	Park et al. (2018)	Cable-driven	Bowden cables	Tensile	1	E
	2018	Jiang et al. (2018)	Pneumatic	Elastomeric	Bending	1	E
	2019	Heung et al. (2019)	Pneumatic	Elastomeric	Rotary	1	F/E
	2019	Shiota et al. (2019)	Pneumatic	Elastomeric	Bending	1	F
	2019	Heung et al. (2019)	Pneumatic	Elastomeric	Bending	1	F
	2019	Zhang et al. (2019a)	Pneumatic	Elastomeric	Stiffening	1	F
f. Thumb	2004	Noritsugu et al. (2004)	Pneumatic	McKibben	Contraction	1	Ab/Ad
	2010	Connelly et al. (2010)	Pneumatic	Textile/fabric	Bending	1	Ad
	2014	Maeder-York et al. (2014)	Pneumatic	Elastomeric	Bending	1	F
	2014	Lee et al. (2014)	Cable-driven	Bowden cables	Tensile	1	Ab/Ad
	2015	Yap et al. (2015a)	Pneumatic	Elastomeric	Bending	1	Ab
	2018	Kim and Park et al. (2018)	Cable-driven	Bowden cables	Tensile	1	Ab/Ad
	2019	Shiota et al. (2019)	Pneumatic	Elastomeric	Bending	1	Ab/Ad

Abbreviations: Ab, abduction; Ad, adduction; E, extension; F, flexion; Su, supination; Pr, pronation.

### 6.1. Head and neck

There are limited studies of soft robotics application above the shoulders. However, there are studies on how soft robotics can help with basic facial motor functions. So far this area of research is minimal, though could pose promise for future studies due to the biomimicry often seen in many SWRs. Due to limited or sufficient data on this particular joint, it is omitted from the final list of considered (real) options.

### 6.2. Shoulder

The shoulder joint is comprised of a combination of DoF and motions that become coupled during certain movements and remain separate entirely for other motions, which create a particularly complex location to provide assistance with wearable devices. The shoulder maintains several rotational DoFs that intersect and do not have perfectly perpendicular axes, as well as another DoF that moves as a translation of the center of the joint itself. This level of complexity has made the shoulder a difficult joint to assist with rigid robots, and many early designs required the user to be seated with their torso fixed in order to utilize the device (Table 1a).

Initial research into SWRs for the shoulder can be observed in the turn of the century, when McKibben actuators were the more common approach (Kobayashi et al. 2007; Balasubramanian et al. 2008). Initial designs were lighter than rigid counterparts and less complex, however, because McKibben muscles could only apply tensile forces the DoF assisted were limited. Significant issues with slippage were observed, and the translation of forces to the human body was not always comfortable for the user (Kobayashi et al. 2007). As cable-driven systems became more common in the 2010s, shoulder assistance with SWRs shifted away from McKibben and fluidic actuators and focused more on using tendons to manipulate the joint (Kesner et al. 2011; Galiana et al. 2012). These systems were far less bulky and conformed more easily to the joint, with less limitation on tensile force or contraction ratio which could support the full RoM, and also facilitate placement to allow support in more DoF (Kobayashi et al. 2007; Galiana et al. 2012). Development for shoulder SWRs became a more active research topic in the later 2010s as NASA began to invest in the “Armstrong,” a wearable suit for astronauts that used tendons to assist the shoulder (Kadivar et al. 2017). It was around the time of this development that increasingly advanced textile actuators emerged in the field (Natividad and Yeow 2016; O’Neill et al. 2017) and McKibben muscles showed more sophisticated and effective designs (Abe et al. 2018). The shoulder has proven to be a difficult joint to assist, and some of the most prevalent SWRs tackling this goal are still seeking to find actuators that most closely resemble human muscle functions and placements (Abe et al. 2019).

### 6.3. Back and trunk

These SWRs are commonly seen to help prevent injuries before they occur when dealing with the back or trunk of the user (Table 1b) and usually focus on posture and lifting assistance (Babicˇ et al. 2017). This is especially critical for factory or industrial workers, or other individuals who do repetitive heavy lifting (Babicˇ et al. 2017; Lamers et al. 2018). Several soft systems utilize passive methods of actuation such as pretensioned elastic bands or pulley systems that help support the back at opportune moments for injury (Cho et al. 2017; Yang et al. 2019). The benefit of actuation that uses tension in-line with the spine is the ability to store and release energy during lifting with the flexion and extension of the trunk. Since the human trunk can be heavy, actuation methods to actively assist movement can be difficult when locating anchor points that do not impede motion or translate forces to become burdensome to other joints.

### 6.4. Elbow

SWRs for elbow assistance range from a focus in rehabilitation to injury prevention (Table 1c). These systems began in a consistent and similar methodology as wearable robots for the shoulder. The initial introduction of the McKibben muscle led to development emerging to design systems in robotics that would facilitate elbow rehabilitation without restricting movement of the user (Noritsugu et al. 2008). McKibben muscles quickly became an issue when translating tensile force to torque at the elbow joint, and with cable-driven systems gaining popularity in LB SWRs, designing cable-driven systems for the UB became a focus for SWRs (Koo et al. 2014). With only a single DoF to maneuver at the elbow, it became more common to integrate designs for assisting both joints simultaneously with underactuation (Abe et al. 2018). Cable-driven systems provide assistance that mimics the biological function of the muscles in the upper arm to enact flexion and extension (Xiloyannis et al. 2017; Chiaradia et al. 2018). By attaching cables to the base of the forearm and routing to an anchor point at the shoulder, the cables can be controlled to provide flexion and extension assistance at the elbow joint (Xiloyannis et al. 2017; Li et al. 2018).

Fluidic actuation approaches specific to the elbow begun to emerge in the 2010s with other pneumatic SWRs and varied stylistically to match the specific need the exosuit is trying to fulfill (Abe et al. 2018). Some used a bellows-type design to push the elbow into extension and pull it into flexion from the armpit area (Oguntosin et al. 2015), while others designed for rotary actuators placed on the exterior side of the elbow joint to apply a rotational torque to induce flexion assistance (Aragane et al. 2008; Noritsugu et al. 2008; Thalman et al. 2018). Elbow assistance is still a topic of high interest. It is less commonly seen as an isolated SWR for the particular joint. The elbow is commonly designed to be assisted as a part of a larger UB system as factors such as posture and weight/load distribution become more critical.

### 6.5. Wrist and hand

SWRs are effective for the human hand due to their ability to easily follow complex motion and utilize the compliance for an underactuated mechanism (Sasaki et al. 2005b). Table 2d–f lists several successful executions of SWRs for assistance to the hand and wrist. Underactuated SWRs use limited inputs for each segment of the hand and allow for reduced computing power and simplified control (Yap et al. 2015b). The goal for these technologies is to restore basic hand movement and functionality to regain independence and achieve ADL (Connelly et al. 2010). There has been a variety of adaptations of soft hand exosuits for rehabilitation over the past decade, and the hand in particular is an assistance location that has drawn a significant amount of attention from the SWR community. Some notable applications of soft robotics on the hand and wrist can be seen as far back as the early 2000s, utilizing the principles of McKibben muscles to modify the existing mechanical behavior to generate new and more helpful actuator performance (Noritsugu et al. 2004; Sasaki et al. 2005b).

Despite major shift of McKibben actuators and expanding capabilities, other avenues of soft actuation were being explored to provide assistance to the human hand. As cable-driven SWRs gained popularity, this strategy actuation is effective as it mimics the natural mechanics of the hand and tendons and often have a low profile, though often requires motors being placed somewhere on the arm or wrist. PAMs (Sasaki et al. 2005b), fluidic elastomers (Polygerinos et al. 2015; Zhao et al. 2016; Jiang et al. 2018; Shiota et al. 2019), and fabric-based inflatable (Noritsugu et al. 2008; Zhu et al. 2017; Realmuto and Sanger 2019) actuation methods have been used to achieve various levels of assistance and are typically all actuated using pneumatics, which pose similar uses of needed tubing and a pneumatic source. Fabric-based pneumatics have shown promise to avoid some of the issues of bulkiness observed in PAMs and elastomers. There is newly emerging research on soft exosuits for the wrist joint with focus on preventative assistance, as well as supporting forearm pronation and supination (Realmuto and Sanger 2019; Park et al. 2019b).

## 7. LB Soft Assistive Robotics

**Figure 6.**
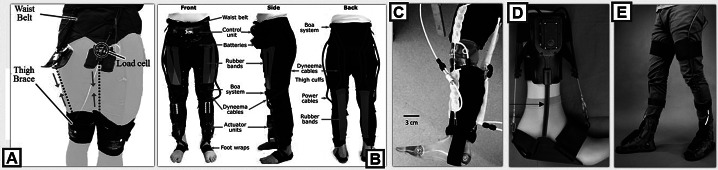
A brief survey of lower body soft wearable robots (SWRs), focused on each joint listed in “UB soft assistive robotics” section. (a) Shows an exosuit developed by the Wyss Institute to support the hip (Lee et al. 2017a). (b) Shows an exosuit with a combination of cables and passive elastic bands used to assist the hip and knee joints (Schmidt et al. 2017). (c) Provides an example of a soft knee exosuit using elastomeric pneumatic artificial muscles (PAMs) (Park et al. 2014b), (d) an ankle device using cable-driven mechanisms to support multiple degrees of freedom from Carnegie Mellon University (Kwon et al. 2019), and (e) shows the ExoBoot, which uses fabric-based inflatables for ankle plantarflexion (Chung et al. 2018).

LB-assistive devices are commonly designed with the purpose of assisting or augmenting the human gait. Assistance is typically aimed at restoring a natural gait pattern to an impaired individual with abnormal gait adaptations. Augmentation is aimed at either increasing walking capabilities for a faster pace or reducing the metabolic cost for walking/running. Using SWRs for LB limbs is a major advancement that allows the user to interface with an LB robot without adding additional weight to their legs (Siviy et al. 2017). Adding weight to the limbs during walking increases the inertia of the limb while in motion which can cause unnatural gait adaptations and safety concerns with balance for increased risk of trips and falls (Browning et al. 2007). In the following sections, each joint will be discussed separately, even if particular devices span across assistance for multiple joints. Figure 6 shows a few of these devices. Tables 3 and 4 provide a comprehensive list of referenced work, specifying the works observed for each section and joint location for assistance.

**Table 3.** This table lists of some of the most cited and prevalent works in soft wearable robots (SWRs) for the lower body over the past decade, focused on the hip and knee joint.

Joint	Year	Soft wearable robot	Actuation	Method	Design	DoF	Motion
g. Hip	2013	Wehner et al. (2013)	Pneumatic	McKibben	Contraction	1	F/E
	2013	Kawamura et al. (2013)	Pneumatic	McKibben	Contraction	1	F
	2015	Asbeck et al. (2015c)	Cable-driven	Bowden cables	Tensile	1	E
	2015	Quinlivan et al. (2015)	Cable-driven	Bowden cables	Tensile	1	E
	2015	Asbeck et al. (2015a)	Cable-driven	Bowden cables	Tensile	1	F
	2015	Wu et al. (2015)	Cable-driven	Bowden cables	Tensile	1	F
	2016	Lee et al. (2016)	Cable-driven	Bowden cables	Tensile	1	F
	2016	Ding et al. (2016)	Cable-driven	Bowden cables	Tensile	1	E
	2017	Schmidt et al. (2017)	Cable-driven	Bowden cables	Tensile	1	E
	2017	Ding et al. (2017)	Cable-driven	Bowden cables	Tensile	1	F
	2017	Karavas et al. (2017)	Cable-driven	Bowden cables	Tensile	1	F
	2018	Thakur et al. (2018)	Pneumatic	Elastomeric	Contraction	1	F
	2018	Poliero et al. (2018)	Cable-driven	Bowden cables	Tensile	1	E
	2019	Di Natali et al. (2019)	Cable-driven	Bowden cables	Tensile	1	E
	2019	Park et al. (2019a)	Cable-driven	Bowden cables	Tensile	1	E
	2019	Panizzolo et al. (2019)	Cable-driven	Bowden cables	Tensile	1	E
	2019	Fraiszudeen and Yeow (2019)	Pneumatic	Textile/fabric	Tensile	1	E
h. Knee	2012	Teng et al. (2012)	Pneumatic	McKibben	Contraction	1	F/E
	2013	Wehner et al. (2013)	Pneumatic	McKibben	Contraction	1	F
	2013	Baiden and Ivlev (2013)	Pneumatic	McKibben	Contraction	1	F
	2014	Park et al. (2014b)	Pneumatic	Elastomeric	Contraction	1	E
	2017	(Schmidt et al. 2017)	Cable-driven	Bowden cables	Tensile	1	E
	2018	Thakur et al. (2018)	Pneumatic	Elastomeric	Contraction	1	E
	2019	(Park et al. 2019a)	Cable-driven	Bowden cables	Tensile	1	E
	2019	Miller-Jackson et al. (2019c)	Pneumatic	Textile/fabric	Rotary	1	E
	2019	Fraiszudeen and Yeow (2019)	Pneumatic	Textile/fabric	Stiffening	1	E
	2019	Ezzibdeh et al. (2019)	Pneumatic	Textile/fabric	Rotary	1	E

Each research group included below has been sorted by the joint assisted and actuation method used.

Abbreviations: Ab, abduction; Ad, adduction; E, extension; F, flexion; Su, supination; Pr, pronation.

**Table 4.** This table is a continuation of the previous table for soft wearable robots (SWRs) for assisting the lower body, detailing work done on the ankle joint.

Joint	Year	Soft wearable robot	Actuation	Method	Design	DoF	Motion
i. Ankle	2006	Gordon et al. (2006)	Pneumatic	McKibben	Contraction	1	P
	2006	Ferris et al. (2006)	Pneumatic	McKibben	Contraction	1	D/P
	2011	Park et al. (2011)	Pneumatic	McKibben	Contraction	2	P, I/E
	2012	Teng et al. (2012)	Pneumatic	McKibben	Contraction	1	D/P
	2012	Wehner et al. (2012)	Cable-driven	Bowden cables	Tensile	1	P
	2013	Asbeck et al. (2013)	Cable-driven	Bowden cables	Tensile	1	P
	2013	Wehner et al. (2013)	Pneumatic	McKibben	Contraction	1	D/P
	2014	Murphy et al. (2014)	Pneumatic	McKibben	Contraction	2	D/P, I/E
	2014	Park et al. (2014a)	Pneumatic	McKibben	Contraction	2	D/P, I/E
	2015	Bae et al. (2015)	Cable-driven	Bowden cables	Tensile	1	D/P
	2015	Low et al. (2015)	Pneumatic	Elastomeric	Contraction	1	D
	2015	Asbeck et al. (2015a)	Cable-driven	Bowden cables	Tensile	1	P
	2016	Low et al. (2016)	Pneumatic	Elastomeric	Contraction	1	D
	2016	Sovero et al. (2016)	Pneumatic	Textile/fabric	Rotary	1	P
	2016	Lee et al. (2016)	Cable-driven	Bowden cables	Tensile	1	D/P
	2017	Awad et al. (2017b)	Cable-driven	Bowden cables	Tensile	1	D/P
	2017	Ding et al. (2017)	Cable-driven	Bowden cables	Tensile	1	P
	2017	Yandell et al. (2017)	Cable-driven	Bowden cables	Tensile	1	P
	2017	Siviy et al. (2017)	Cable-driven	Bowden cables	Tensile	1	P
	2017	Karavas et al. (2017)	Cable-driven	Bowden cables	Tensile	1	P
	2017	Hong et al. (2017)	Pneumatic	McKibben	Contraction	1	P
	2017	Bowers et al. (2017)	Pneumatic	McKibben	Contraction	1	D/P
	2018	Chung et al. (2018)	Pneumatic	Textile/fabric	Stiffening beam	1	P
	2018	Iwata et al. (2018)	Pneumatic	McKibben	Contraction	1	D
	2019	Thalman et al. (2019)	Pneumatic	Textile/fabric	Contraction	1	D
	2019	Di Natali et al. (2019)	Cable-driven	Bowden cables	Tensile	1	D/P
	2019	Kwon et al. (2019)	Cable-driven	Bowden cables	Tensile	1	D/P
	2019	Zhang et al. (2019b)	Pneumatic	McKibben	Contraction	1	D/P
	2020	Thalman et al. (2020)	Pneumatic	Textile/fabric	Contraction	1	P
	2020	Thalman and Lee (2020)	Pneumatic	Textile/fabric	Stiffening beam	1	I/E

This is another heavily researched area and the worked cited below are some of the most well-known and exemplary works sorted by the assisted degree of freedom and actuation method used.

Abbreviations: D, dorsiflexion; E, eversion; I, inversion; P, plantarflexion.

### 7.1. Hip

The human hip is a critical joint that allows for the transmission of force from the grown to the torso. The hips also assist in standing, balancing, postural, and sitting tasks, and hip joint actuation with SWRs (shown in Table 3g) primarily relies on cable-driven actuation, due to the high torque output from the tension forces of the cables as shown in (Wu et al. 2015; Asbeck et al. 2015c; Ding et al. 2016; Lee et al. 2017a; Ding et al. 2018; Di Natali et al. 2019), though recent work has shown how fluidic actuators done by groups (Kawamura et al. 2013; Thakur et al. 2018) and passive actuation methods seen in (Lee et al. 2017b) can be used in hip assistance. Hip assistance from SWRs can be used for both stationary sit-to-stand tests or during corrective gait therapy (Abouhossein et al. 2018; Fraiszudeen and Yeow 2019).

### 7.2. Knee

SWRs have been designed to help assist in both knee flexion and extension, as well as to provide stability to the knee joint to prevent buckling at the knee as shown in Table 3h. SWRs for the knee are often seen as stationary systems designed to be worn to address issues with gait symmetry (Elliott et al. 2013). This can be done by assisting the knee in either flexion or extension for executing the swing phase or providing support during the weight shift in the stance phase (Teng et al. 2012). Many designs are cable-driven systems that pull the knee into both flexion and extension (Asbeck et al. 2013; Di Natali et al. 2019). Other research has been done on using fluidic actuators for knee assistance (Teng et al. 2012; Park et al. 2014b; Ezzibdeh et al. 2019; Miller-Jackson et al. 2019c). Issues with pneumatic knee assistance are found in the latency in the actuators firingas mentioned in reference to pneumatic control. Other methods of actuation include passive elastic elements to provide preventative microcorrections (Elliott et al. 2013).

### 7.3. Ankle and foot

Prior to advancements in portable, wearable robotics, most ankle rehabilitation robots focused on seated therapies on robotic platforms that would adjust or orient the ankle into various positions (Table 4i). Designing wearable robots for the ankle joint has been difficult until recent years, as rigid robots can be heavy and adding excessive weight to the ankle joint can lead to increased risk of trips or slowed gait due to higher inertia at the foot while walking (Browning et al. 2007). The ankle joint has two major degrees of freedom, the dorsiflexion/plantarflexion (DP) axis and the inversion/eversion (IE) axis (Hong et al. 2017; Perez-Ibarra et al. 2017). During walking, the DP axis plays a major role in assisting in forward locomotion and foot ground clearance (toe up and toe down), while IE assists in ankle stability and resilience to external perturbations during movement (lateral ankle rotation) (Hong et al. 2017; Siviy et al. 2017).

A few early instances of soft actuators seen for ankle rehabilitation consisted of a McKibben actuator affixed to a rigid orthosis. Issues with latency and weight were still prevalent, however, and as more opportunities became available with DARPA and other funding sources, there was a noticeable shift toward one of the most common approaches for SWRs for the ankle: Cable-driven systems or other forms of contracting actuation methods have been widely seen in use ankle assistance, likely due to similarities with the tendon and muscle groups that control the foot (Park et al. 2011; Ren et al. 2017; Iwata et al. 2018). This can be attributed to the high complexity and torque requirements for the ankle joint, specifically plantarflexion, during walking (Teng et al. 2012; Murphy et al. 2014; Chung et al. 2018). There is extensive research on using cable-driven systems to assist ankle function, both through calculated torque generation and metabolic cost reduction experimentation (Chin et al. 2009; Chung et al. 2018; Yandell et al. 2019). Cable-driven ankle SWRs tend to switch between both assistance and augmentation, with applications ranging from warfighter assistance to poststroke rehabilitation during walking (Bae et al. 2015; Sovero et al. 2016; Siviy et al. 2017; Kwon et al. 2019). Fluidic actuation methods for the ankle are relatively popular, with some of the most notable contributions using PAM Mckibben-type actuators (Wehner et al. 2012; Murphy et al. 2014; Sovero et al. 2016; Bowers et al. 2017), with a select few fabric-based inflatable devices (Chung et al. 2018; Thalman et al. 2019; Thalman and Lee 2020). Some applications seek simply to add stability and prevent trips and falls using the user’s body weight to distribute pressure through different chambers based on where the user is applying their body weight to their foot (Babu et al. 2018).

## 8. Discussion

This review presents an examination of SWR devices for human assistance. A survey of actuation methods used in wearable devices, fabrication advancements, and applications is presented to provide a comprehensive overview of trends with the field. Soft robotics is an effective approach to providing wearable robotic technologies with lightweight, low cost, and comfortable adaptations, while pushing the boundaries of material science and sensing technology. The rapidly growing field is challenging the way that roboticists have thought about wearable assistive devices and is providing new pathways to enabling users to have wearable assistive robotics that are nearly transparent to their movement.

Soft robotic actuators have many benefits; however, there are areas where improvements are still needed to further enhance the field. Potential areas for growth and current issues that are seen across most wearable robots in general are the power sources for actuation. Pneumatic systems require some method of obtaining pressurized fluid to perform actuation, which is usually done by tethering the SWR to a compressor and regulating the resulting output pressure (Sasaki et al. 2005b; Ding et al. 2014; Wang 2016). The ideal system would be a standalone electropneumatic system, giving the user unconstrained mobility (Manfredi and Cuschieri 2019); however, portable pneumatic sources typically have struggled to provide sufficient flow rate or volume for most adult users without excessive weight (Wehner et al. 2012; Thalman et al. 2018). Latency issues resulting from pneumatic power sources is common in soft robotics (Malcolm et al. 2013). This can be mitigated somewhat with the higher flow rates that can be achieved from directly connecting to air supply lines in existing infrastructure or tubing with larger diameter (Ding et al. 2014). Cable-driven systems use battery-powered motors and control boards to induce actuation, which can be easily implemented into a hip-mounted pouch or a backpack to house all control components (Bae et al. 2015). Actuation motors are commonly mounted on the body to ensure the cables remain routed along the path needed to produce desired torques. While advantageous to have all hardware easily portable, a disadvantage is that the weight is often borne by the user.

The compliant and extremely variable positioning and behaviors of materials used in many SWRs can cause difficulties when higher levels of flexibility are introduced to the core foundation of the device. Control of compliant actuation methods is usually based on oversimplified models based on pressure, volume, simplified geometries, constant-curvature, or basic deformation (Petersen and Shepherd 2018). While cable-driven system control has been more widely investigated, control of fluidic or extremely compliant actuator bodies has created interest in the soft robotics community posed as a free-form, open-ended question: How can soft-bodied actuators be controlled? Open loop control is often times not sufficient, and so closed loop control is attempted using these simplified models. Bang-bang control is a common control strategy, using pressure or bending/curvature sensors to trigger pressurization at defined intervals (Petersen and Shepherd 2018). Modern sensing technology is shifting toward integrating sensing capabilities into fabric-based actuators or into the fabric itself. By mimicking clothing, these garment-like sensors are less cumbersome and invasive to the user. Strain-gauge goniometers and flexible resistive sensors can be used to measure the body’s displacement directly and are a popular method of collecting data of actuator positioning and behavior (Mengüç et al. 2014; Park et al. 2014a; Porciuncula et al. 2018; Kim et al. 2019). Incorporating Inertial Measurement Units (IMUs) is another common approach to collecting accurate data on the real-time positioning of a specified point on the user or exosuit (Karavas et al. 2017; Abouhossein et al. 2018). Muscle activity can be also used to trigger the wearable device at certain intervals where surface mount electromyography (sEMG) sensors are used to monitor the electrical potential of the user’s muscles (Ferris et al. 2006; Porciuncula et al. 2018). Force-sensitive resistors (FSRs) are used frequently for LB wearables devices and can be embedded in soft silicone insoles worn by the user to track weight distribution on certain regions of the foot (Poliero et al. 2018; Porciuncula et al. 2018).

Soft robotics poses as a safe method to provide robotic assistance to individuals who may have unpredictable behaviors or movements patterns, without a noticeable amount of weight or bulk (Polygerinos et al. 2017). LB-assistive robotics are typically focused on gait training, restoring some form of symmetry to an otherwise abnormal gait pattern, or offloading muscle effort for a gait with reduced strain to the user (Kawamura et al. 2013). These exosuits can be used to actively prevent compensatory gait mechanics, provide additional support and stiffness, or encourage proper gait mechanics (Abouhossein et al. 2018). Some assistive devices, especially those focused on UB systems and the hand, aim to help impaired user regain independence and achieve ADL (Morales et al. 2011; Maciejasz et al. 2014). With preventative assistance, SWRs alleviate strain to particular parts of the body by offloading or translating pressure and stress from the user. This can help prevent injuries caused by heavy lifting, repetitive tasks, and poor ergonomics. Human augmentation can be achieved with wearable devices by providing high amounts of torque and force, while some SWRs can assist users carrying extremely heavy loads over long distances, lift at higher capacities, run faster with a lower metabolic cost, and even increase sports performance (Roam 2009; Lee et al. 2017a).

Review of existing SWRs presented in this manuscript shows a trend that is beginning to favor actuator designs that can be easily embedded into the user’s clothing with minimal interference to normal RoM and increased comfort. Observations from current devices and prevalence in the field based on publication records suggest that assistive soft robots are still in their infancy. This leaves ample opportunity for the field to begin to further explore how fabric-based actuators can be integrated together with other efforts toward functional garments to advance the way that rehabilitative robotics is approached for UB and LB assistance.
